# Supplementary material for: Major sea level fall during the Pliocene M2 glaciation
Source: Nat Commun. 2025 Aug 16;16:7641. doi: 10.1038/s41467-025-62446-x (PMC12357856; doi:10.1038/s41467-025-62446-x)
Supplement: Supplementary file 1 — Supplementary Information [file 41467_2025_62446_MOESM1_ESM.pdf]

Supplementary Information

Possible contamination of benthic foraminiferal Mg/Ca indicated by Mn/Ca

In Braaten *et al.* (2023), *O. umbonatus* Mn/Ca values are an order of magnitude higher than ideal values, with a mean of 1050 μmol/mol at ODP Site 849 and 1072 μmol/mol at IODP Site U1308 (Fig. S4a, b)[1]. Mn can be present in many different phases on foraminiferal tests, and high Mn/Ca values are not always a reliable indicator for contamination of Mg/Ca. Furthermore, Braaten *et al.* (2023) point out that their highest Mg/Ca values do not correspond with maximum Mn/Ca values, and there is no significant correlation on a Mg/Ca-Mn/Ca cross-plot[1]. However, their high Mg/Ca values have the appearance of fliers in a noisy record, which could be caused by other factors, and which would mask any underlying correlation between Mg/Ca and Mn/Ca on a simple cross-plot. We recommend that Mn/Ca records are shown as down-core records instead of cross plots. On a downcore record, at ODP Site 849 *O. umbonatus* Mn/Ca decreases ~40% between 3.33 and 3.30 Ma, and subsequently increases between 3.30 and 3.29 Ma at ODP Site 849 (Fig. S4a). The similarity of this overall trend with the Mg/Ca record is not necessarily an issue, but it does raise the possibility that the degree of cooling during the M2 glaciation has been overestimated.

At ODP Site 982, Mn/Ca are from 36-131 μmol/mol, with an average of 79 μmol/mol, which is acceptable[2], and the overall trends of Mg/Ca and Mn/Ca are obviously different (Fig. S4c, e). However, at ODP Site 1241, *Melonis* Mn/Ca are also higher than ideal, with a mean of 460 μmol/mol, and generally increased from 3.313 Ma to 3.184 Ma. In the Material and Methods, we described the reason why we consider the effect of relatively high Mn/Ca ratio on Mg/Ca records at ODP Site 1241 is limited and omit reductive step in this study. If we assume the authigenic coatings at ODP Site 1241 have a Mg/Mn ratio of 0.2 mol/mol[3], a mass balance approach to correct the Mg/Ca values reduces Mg/Ca by ~0.1 mmol/mol, but does not significantly affect the overall trends or amplitude of change (Fig. S4f). Therefore, we show only raw Mg/Ca values in the main text, and use these in our calculations. We also applied the same Mn correction on

ODP Site 849 Mg/Ca, and it reduced Mg/Ca by ~0.2-0.3 mmol/mol. The offsets between pre/post M2 and M2 maximum also decreased ~0.1 mmol/mol (Fig. S4f).

Melonis spp. δ^{13}C and B/Ca reflect porewater chemistry rather than bottom water

Benthic foraminiferal δ^{13}C has the potential to indicate changes in bottom water mass, and B/Ca can reflect carbonate saturation state, which could influence Mg/Ca records in some foraminifera species. We present epifaunal δ^{13}C and *Melonis* spp. δ^{13}C and B/Ca records, even though we find Mg/Ca in *Melonis* spp. calcite shells to be insensitive to microhabitat ΔCO_3^{2-}. *Melonis* δ^{13}C ($\delta^{13}C_M$) reaches a minimum during MIS M2, with amplitudes of ~1.2‰ and ~0.4‰ at ODP Sites 982 and 1241 respectively, which could be interpreted as a change in bottom water chemistry at first sight (Fig. S5). However, we instead attribute this change to a change in porewater chemistry, since the *C. wuellerstorfi* δ^{13}C records, which were thought to be recording bottom water $\delta^{13}C_{DIC}$ signals do not show the same signal as our infaunal δ^{13}C records, and relatively stable offsets existed between *C. wuellerstorfi* δ^{13}C records at ODP Sites 982 and 1241, while the offsets of *Melonis* are variable (Fig. 3, S5)[4,5]. The reason as indicated by the cross correlation between porewater and bottom water $\delta^{13}C_{DIC}$ of the modern oceans is, the bottom water $\delta^{13}C_{DIC}$ values of Atlantic stations is heavier than that of Pacific stations, while the porewater $\delta^{13}C_{DIC}$ is independent to bottom water (Fig. S6). The decrease in $\delta^{13}C_M$ in our downcore records is also associated with a decrease in *Melonis* spp. B/Ca, with amplitudes of around 20 µmol/mol and 10 µmol/mol at ODP Sites 982 and 1241 respectively, reflecting a reduction in porewater ΔCO_3^{2-} during the M2 glaciation. We suggest that this was potentially caused by enhanced export productivity in a cooler climate, leading to enhanced lowering of carbonate saturation state in the porewaters relative to bottom waters (Fig S5)[6,7]. Therefore, the lower *Melonis* B/Ca at ODP Site 1241 compared to ODP Site 982 indicate that the porewaters at ODP Site 1241 were less well saturated with respect to calcium carbonate. In the modern ocean, porewater ΔCO_3^{2-} has linear relationship with bottom water[8], and using pH and Alk values from GLODAP datasets, the calculated ΔCO_3^{2-} using 'seacarb package' [9] at Sites

982 and 1241 are 55.7 μmol/kg and 5.5 μmol/kg at ODP Sites 982 and 1241 respectively. In addition, focusing on the second (~3.291-3.282 Ma) $\delta^{18}O_{SW}$ increase at both sites, we note that it is short-lived and does not correspond to any transient shift in epifaunal $\delta^{13}C$, although it is associated with a rapid BWT warming at each site (Fig. S5).

Table S1

Location, water depth and present BWT of studied sites

Region	Site	Lat<br>°N	Lon<br>°E	Depth<br>m	BWT<br>℃
North Atlantic	ODP 982	57.52	-15.87	1134	~ 6
Eastern Pacific	ODP 1241	5.84	-86.44	2027	~ 2
Norwegian Sea	KN177-2 MC4	62.12	2.72	418	5.15
Norwegian Sea	KN177-2 MC6	62.60	1.74	695	-0.47
Norwegian Sea	KN177-2 MC11	63.03	0.81	1285	-0.80
Norwegian Sea	KN177-2 MC14	62.82	1.30	965	-0.74
Sulawesi Margins	BJ8-03 MC24	-5.06	117.45	832	5.18
Little Bahama Bank	OC205-2 BC77	26.23	-77.66	433	16.55

Table S2 Results of with and without reductive step cleaning test

Sample Label	Mg/Ca (mmol/mol) Without reductive	Mg/Ca (mmol/mol) With reductive	Mn/Ca (μmol/mol) Without reductive	Mn/Ca (μmol/mol) With reductive
162-982B-12H-1W,95-99	2.05	1.97	59.5	51.7
162-982B-12H-1W,103-107	1.92	1.85	68.9	60.8
162-982B-12H-1W,118-122	1.92	1.84	75.8	65.9
162-982B-12H-1W,134-138	1.94	1.94	73.3	65.0
162-982B-12H-2W,101-105	1.98	1.85	63.8	60.9
162-982B-12H-3W,61-65	1.93	1.80	44.7	45.2
162-982B-12H-3W,69-73	1.94	1.76	44.5	37.7
202-1241B-12H-3W,22-25	1.46	1.39	191.	160
202-1241B-12H-3W,32-35	1.49	1.46	206	159
202-1241B-12H-3W,42-45	1.40	1.50	233	172
202-1241B-12H-3W,57-60	1.39	1.32	211	175
202-1241B-12H-3W,64-67	1.43	1.36	220	164
202-1241B-12H-3W,82-85	1.40	1.28	221	192
202-1241C-5H-5W,134-137	1.17	1.20	508	345
202-1241C-5H-6W,5-8	1.17	1.12	500	305
202-1241C-5H-6W,34-37	1.22	1.20	459	302
202-1241C-5H-6W,44-47	1.12	1.04	381	337

202-1241C-5H-6W,54-57	1.03	0.97	383	270
202-1241C-5H-6W,64-67	1.04	1.03	328	229

Figure S1 Average values of porewater ΔCO_3^{2-} (sediment depth of 0-10 cm) versus bottom water ΔCO_3^{2-} from Atlantic stations (same stations in Fig. 2)[8].

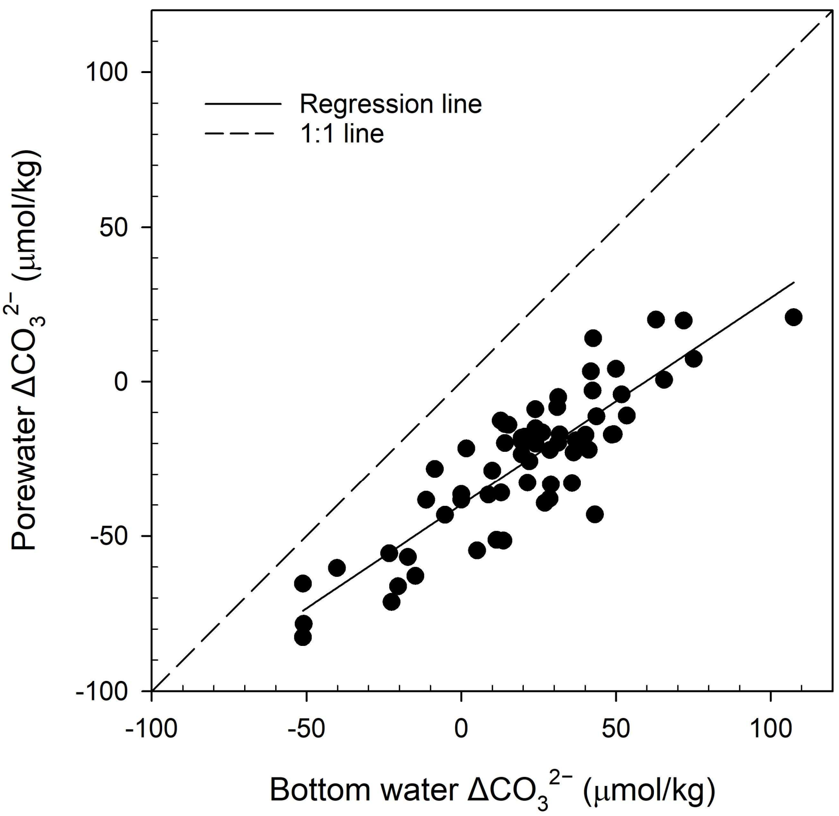

Figure S2 Core-top foraminiferal B/Ca ratios versus bottom water ΔCO_3^{2-}. Data are from Rae et al., 2011[10], Yu et al., 2007[11] and this study. Violet triangles – *C. wuellerstorfi*; Orange squares – Epifaunal species except *C. wuellerstorfi*; Teal open circles – *Melonis* spp.; Teal solid circles – Infaunal species except *Melonis*.

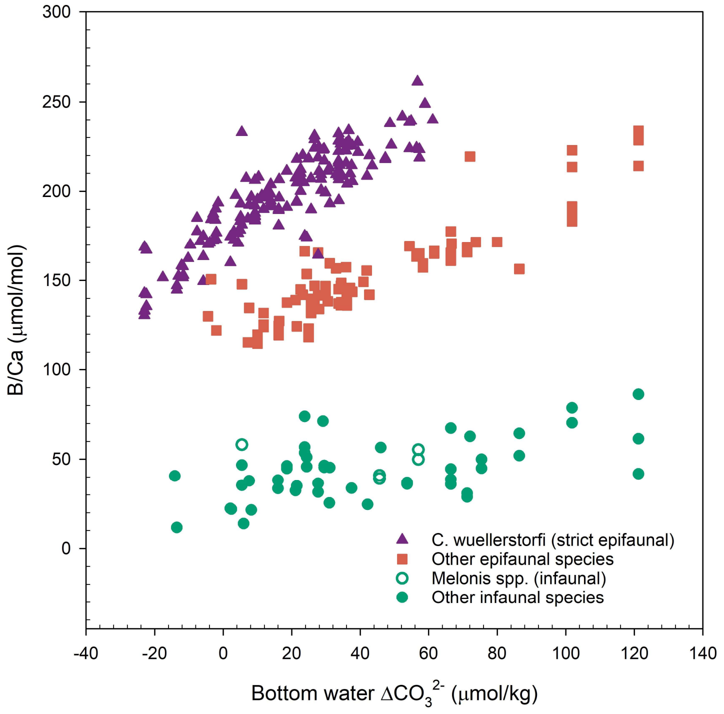

Fig S3 a. Map of sites used in this study (ODP Sites 982 and 1241, indicated by red dots) and all other sites discussed in the main text (ODP Sites 849, 999,1267 and IODP Sites U1308, U1313, indicated by blue dots) and schematic pathways and the sources of modern Atlantic bottom water (adjusted from Kirby *et al.*, 2020; Liu and Tanhua, 2021[12,13]. Blue arrows indicate the source and pathways of North Atlantic Deep Water (NADW), the origin of which is Labrador Sea Water (LSW), the Iceland–Scotland Overflow Water (ISOW) and the Denmark Strait Overflow Water (DSOW). Grey arrows indicate the source and pathways of Antarctic Bottom Water (AABW), the origin of which is Circumpolar Deep Water (CDW) and Weddell Sea Bottom Water (WSBW); b. Selected transect in Atlantic to show the vertical distribution of the main water masses above the studied and discussed sites. Light Grey dots indicate the locations of the World Ocean Circulation Experiment (WOCE) CTD stations[14], red lines highlight the selected transect; c. Section plots of salinity along the selected transect shown in the panel b (salinity data from Schlitzer, 2000[14]). Red stars mark the location of the sites, yellow letters mark the distribution of water masses[13]. In the modern ocean, IODP Site U1313 is bathed by 100% NADW, ODP Site 1267 is bathed by a mixture of 70-80% NADW and 20-30% AABW[12].

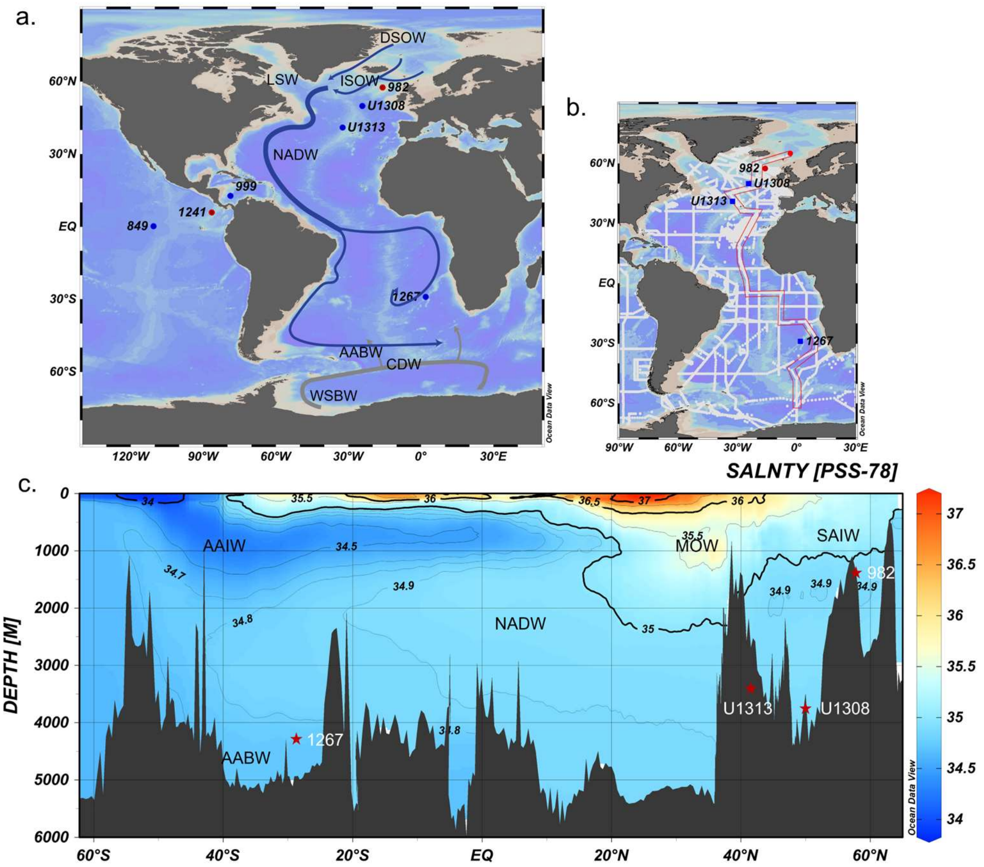

Figure S4 Comparison of Mg/Ca (red) and Mn/Ca (blue) ratios of *O.umbonatus* and *Melonis* spp. records for two intervals in the Pliocene. a. *O.umbonatus* across the M2 glaciation at ODP Site 849[1]. b. *O.umbonatus* across the M2 glaciation at IODP Site U1308[1]. c. *Melonis* spp. across the M2 glaciation at ODP Site 982. d. *Melonis* spp. across MIS CN5 at ODP Site 982. e. *Melonis* spp. across the M2 glaciation at ODP Site 1241. f. Comparison of Mg/Ca records before (violet) and after (teal) Mn-coating correction at ODP Sites 1241 and 849.

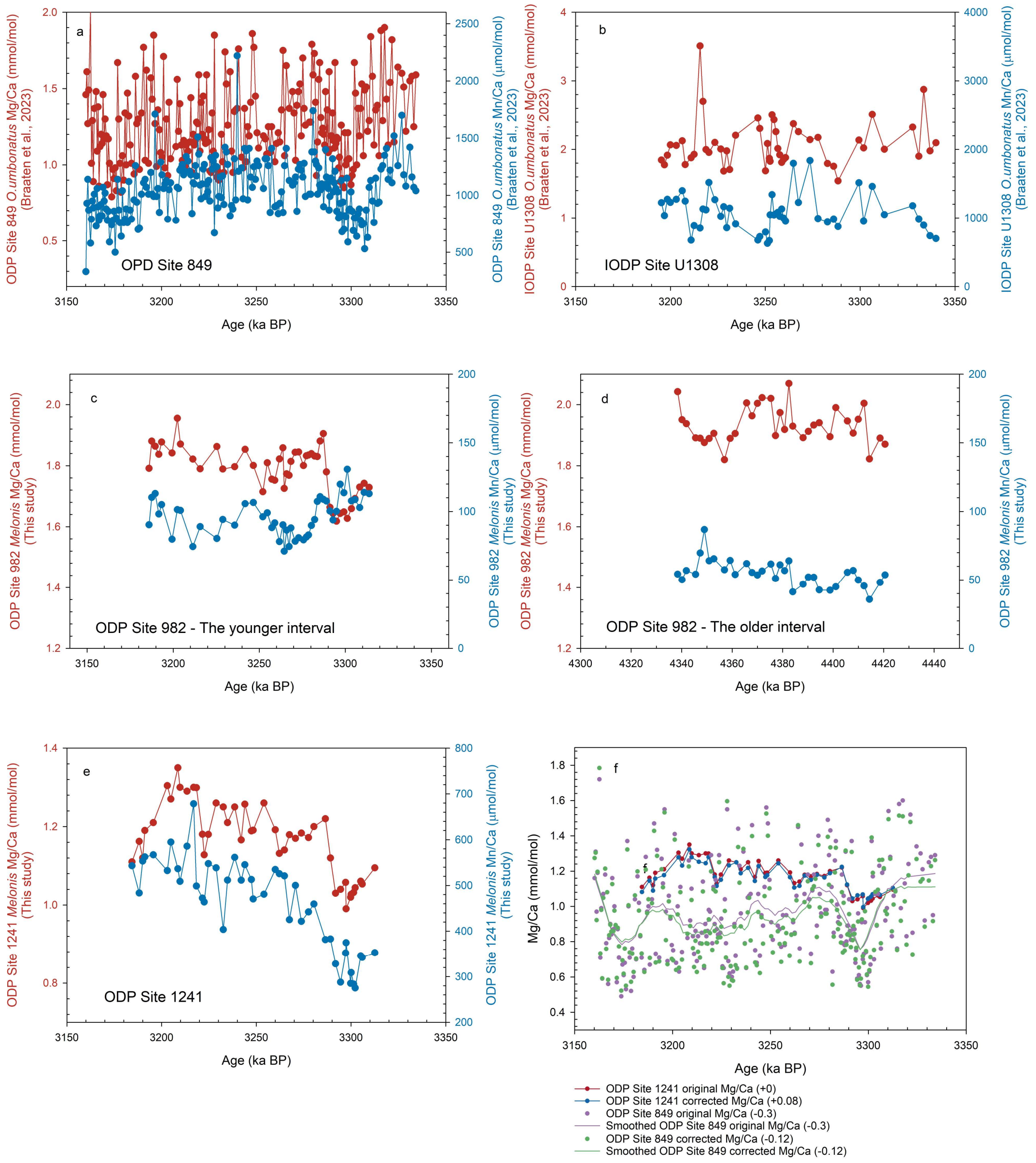

Figure S5 *Melonis* spp. δ^{13}C and B/Ca compared with epifaunal δ^{13}C and productivity records at: a. ODP Site 982: Black circles - δ$^{13}C_M$; Violet circles - B/Ca; Black line - epifaunal δ^{13}C[4]; Teal line - alkenone mass accumulation rate at ODP Sites 982[6]. b. ODP Site 1241 and nearby stations: Black circles - δ$^{13}C_M$; Violet circles - B/Ca; Black line - epifaunal δ^{13}C[5]; Light blue line - alkenone mass accumulation rate at ODP Site 849[7]; Blue line - alkenone mass accumulation rate at IODP Site U1338[7].

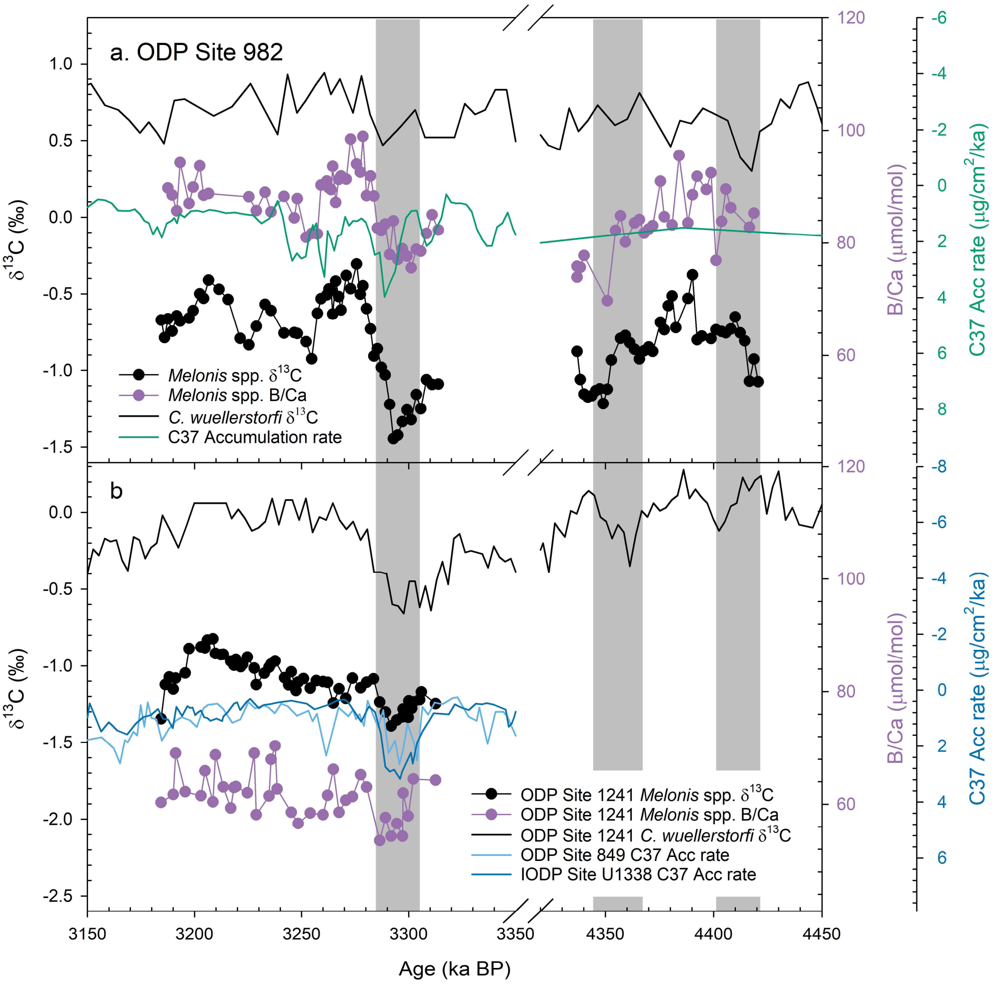

Figure S6 Average values of porewater $\delta^{13}C_{DIC}$ (0-10 cm) versus bottom water $\delta^{13}C_{DIC}$, original data from [15-18]. Green circles – Pacific; Violet triangles – Atlantic

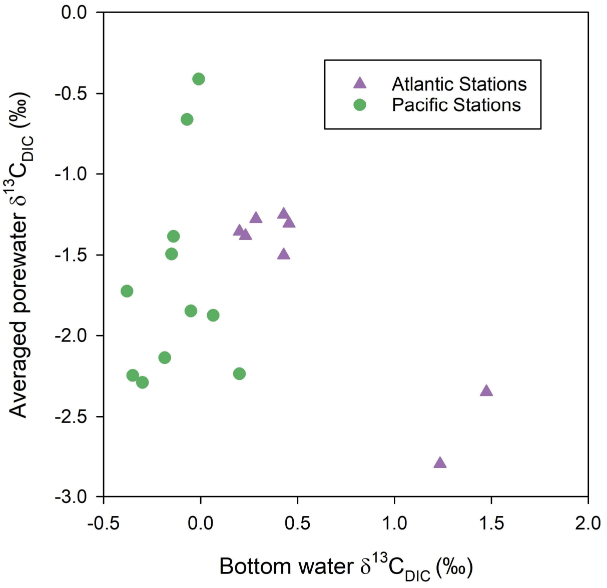

Figure S7 Sampling resolution of Mediterranean planktonic foraminiferal $\delta^{18}O$ record at MIS M2. Red circles- $\delta^{18}O_{SW}$ record at ODP Site 982; Blue diamonds - $\delta^{18}O_{SW}$ record at ODP Site 1241; Violet line - Inferred records based on Mediterranean sea level estimate[19]; Black circles - Mediterranean planktonic foraminiferal $\delta^{18}O$ record, which was used to reconstruct in Mediterranean sea level change in Rohling *et al.* (2014)[20]. Grey band shows the second phase of glaciation, which we suggest has not been captured in Rohling *et al.* (2014).

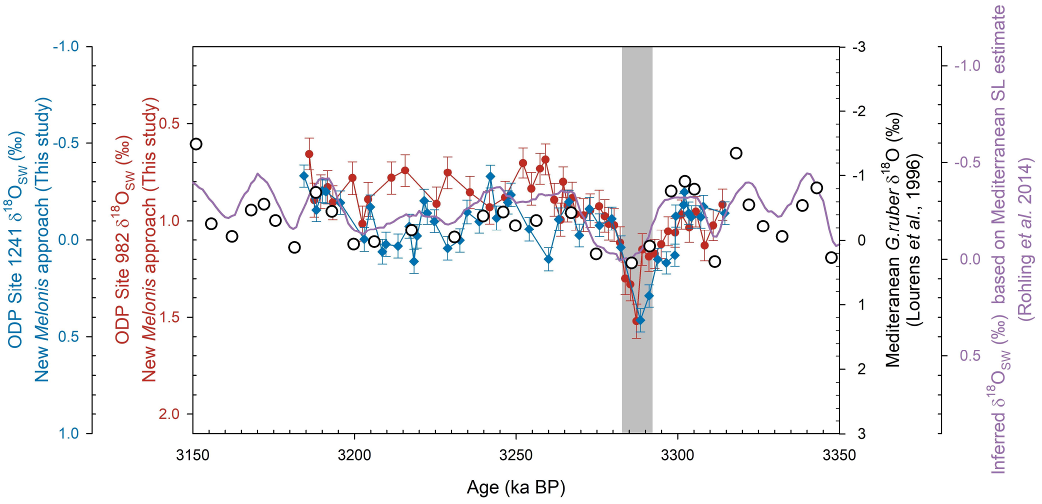

Figure S8 $\delta^{18}O$ of benthic foraminifera stack and from sites used in Fig. 5 and Fig. 6. Black – LR04 stack[21]; Pink – *Melonis* from ODP Site 982 (this study); Orange – *Melonis* from ODP Site 1241 (this study); Green – *C. wuellerstorfi* from ODP Site 1267[12]; Blue - *C. wuellerstorfi* from IODP Site 1331[12]; Light blue - *C. wuellerstorfi* from ODP Site 999[22]. The numbers in the legends indicate the constant values put on the records of each site relative to LR04 stack to make them on the same scale.

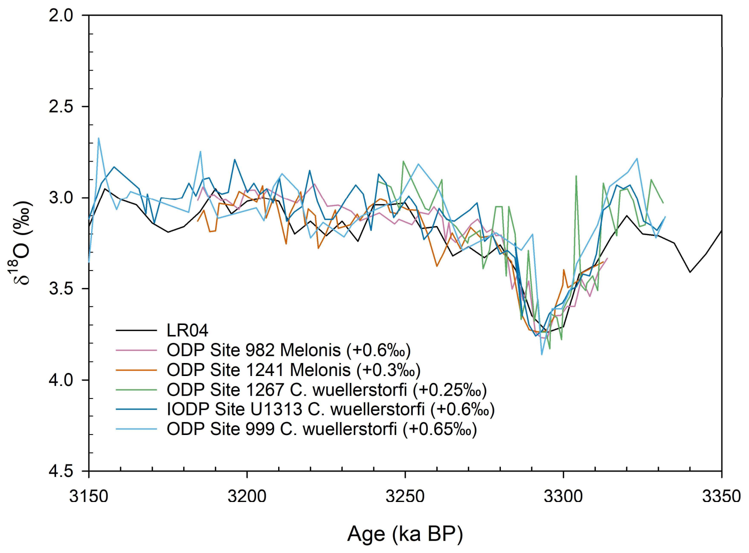

Supplementary References

1 Braaten, A. H. *et al.* Limited exchange between the deep Pacific and Atlantic oceans during the warm mid-Pliocene and Marine Isotope Stage M2 "glaciation". *Clim. Past* **19**, 2109-2125, doi:10.5194/cp-19-2109-2023 (2023).

2 Boyle, E. A. Manganese carbonate overgrowths on foraminifera tests. *Geochim. Cosmochim. Acta* **47**, 1815-1819, doi:https://doi.org/10.1016/0016-7037(83)90029-7 (1983).

3 Hasenfratz, A. P. *et al.* Determination of the Mg/Mn ratio in foraminiferal coatings: an approach to correct Mg/Ca temperatures for Mn-rich contaminant phases. *Earth Planet. Sci. Lett.* **457**, 335-347, doi:https://doi.org/10.1016/j.epsl.2016.10.004 (2017).

4 Hodell, D. A. & Venz-Curtis, K. A. Late Neogene history of deepwater ventilation in the Southern Ocean. *Geochem. Geophys. Geosyst.* **7**, Q09001, doi: https://doi.org/10.1029/2005GC001211 (2006).

5 Steph, S. *Pliocene stratigraphy and the impact of Panama uplift on changes in Caribbean and tropical East Pacific upper ocean stratification (6-2.5 Ma)-Pliozäne Stratigraphie und der Einfluss des Panama-Seeweges auf die Stratifizierung der Oberflächen-Wassermassen in der Karibik und im tropischen Ost-Pazifik (6-2.5 MA)*, Christian-Albrechts-Universität, (2005).

6 Bolton, C. T., Lawrence, K. T., Gibbs, S. J., Wilson, P. A. & Herbert, T. D. Biotic and geochemical evidence for a global latitudinal shift in ocean biogeochemistry and export productivity during the late Pliocene. *Earth Planet. Sci. Lett.* **308**, 200-210, doi:https://doi.org/10.1016/j.epsl.2011.05.046 (2011).

7 Kimble, K., Herbert, T. & Jones, C. Pliocene weakening of gradients in temperature but not in productivity in the eastern equatorial Pacific. *Paleoceanogr. Paleoclimatol.* **39**, e2023PA004711, doi:https://doi.org/10.1029/2023PA004711 (2024).

8 Weldeab, S., Arce, A. & Kasten, S. Mg/Ca-$\Delta CO_{3porewater}^{2-}$–temperature calibration for Globobulimina spp.: A sensitive paleothermometer for deep-sea temperature reconstruction. *Earth Planet. Sci. Lett.* **438**, 95-102, doi:https://doi.org/10.1016/j.epsl.2016.01.009 (2016).

9 Gattuso, J.-P., Epitalon, J.-M., Lavigne, H. & Orr, J. Seacarb: Seawater carbonate chemistry. R package version 3.3.0., doi:http://CRAN.R-project.org/package=seacarb (2021).

10 Rae, J. W. B., Foster, G. L., Schmidt, D. N. & Elliott, T. Boron isotopes and B/Ca in benthic foraminifera: Proxies for the deep ocean carbonate system. *Earth Planet. Sci. Lett.* **302**, 403-413, doi:https://doi.org/10.1016/j.epsl.2010.12.034 (2011).

11 Yu, J. & Elderfield, H. Benthic foraminiferal B/Ca ratios reflect deep water carbonate saturation state. *Earth Planet. Sci. Lett.* **258**, 73-86, doi:https://doi.org/10.1016/j.epsl.2007.03.025 (2007).

12 Kirby, N. *et al.* On climate and abyssal circulation in the Atlantic Ocean during late Pliocene marine isotope stage M2, ~3.3 million years ago. *Quat. Sci. Rev.* **250**, 106644, doi:https://doi.org/10.1016/j.quascirev.2020.106644 (2020).

13 Liu, M. & Tanhua, T. Water masses in the Atlantic Ocean: Characteristics and distributions. *Ocean Sci.* **17**, 463-486, doi:10.5194/os-17-463-2021 (2021).

14 Schlitzer, R. Electronic atlas of WOCE hydrographic and tracer data now available. *Eos, Transactions American Geophysical Union* **81**, 45-45, doi:https://doi.org/10.1029/00EO00028 (2000).

15 McCorkle, D. C. & Emerson, S. R. The relationship between pore water carbon isotopic composition and bottom water oxygen concentration. *Geochim. Cosmochim. Acta* **52**, 1169-

1178, doi:https://doi.org/10.1016/0016-7037(88)90270-0 (1988).

16 McCorkle, D. C., Emerson, S. R. & Quay, P. D. Stable carbon isotopes in marine porewaters. *Earth Planet. Sci. Lett.* **74**, 13-26, doi:https://doi.org/10.1016/0012-821X(85)90162-1 (1985).

17 McCorkle, D. C. & Klinkhammer, G. P. Porewater cadmium geochemistry and the porewater cadmium:δ13C relationship. *Geochim. Cosmochim. Acta* **55**, 161-168, doi:https://doi.org/10.1016/0016-7037(91)90408-W (1991).

18 Papadimitriou, S., Kennedy, H. & Thomas, D. N. Rates of organic carbon oxidation in deep sea sediments in the eastern North Atlantic from pore water profiles of O_2 and the $\delta^{13}C$ of dissolved inorganic carbon. *Mar. Geol.* **212**, 97-111, doi:https://doi.org/10.1016/j.margeo.2004.08.003 (2004).

19 Rohling, E. J. *et al.* Sea-level and deep-sea-temperature variability over the past 5.3 million years. *Nature* **508**, 477-482, doi:10.1038/nature13230 (2014).

20 Lourens, L. J. *et al.* Evaluation of the Plio-Pleistocene astronomical timescale. *Paleoceanography* **11**, 391-413, doi:https://doi.org/10.1029/96PA01125 (1996).

21 Lisiecki, L. E. & Raymo, M. E. A Pliocene-Pleistocene stack of 57 globally distributed benthic δ18O records. *Paleoceanography* **20**, PA1003, doi:https://doi.org/10.1029/2004PA001071 (2005).

22 de la Vega, E., Chalk, T. B., Wilson, P. A., Bysani, R. P. & Foster, G. L. Atmospheric CO2 during the Mid-Piacenzian Warm Period and the M2 glaciation. *Sci. Rep.* **10**, 11002, doi:10.1038/s41598-020-67154-8 (2020).
